# Dual Pathway Inhibition in Patients with Atherosclerosis with or without Heart Failure: Insights from the XATOA Registry

**DOI:** 10.1016/j.cjco.2025.01.013

**Published:** 2025-01-22

**Authors:** Pishoy Gouda, Justin A. Ezekowitz, Alain Gay, Kai Vogtländer, Victor Aboyans, Sebastian Debus, Keith Fox, Uwe Zeymer, Robert Welsh

**Affiliations:** a Department of Medicine, University of Alberta and Mazankowski Alberta Heart Institute, Edmonton, Alberta, Canada; b Canadian VIGOUR Center, Edmonton, Alberta, Canada; c Bayer AG, Berlin, Germany; d Bayer AG, Wuppertal, Germany; e Department of Cardiology, Dupuytren University Hospital, Limoges, France; f Department of Vascular Medicine, Vascular Surgery, Angiology, Endovascular Therapy, University of Hamburg-Eppendorf, Hamburg, Germany; g Centre for Cardiovascular Science, University of Edinburgh, Edinburgh, United Kingdom; h Institut für Herzinfarktforschung, Ludwigshafen am Rhein, Germany

## Abstract

**Background:**

Patients with atherosclerotic cardiovascular disease might benefit from dual pathway inhibition (DPI) therapy, which includes rivaroxaban and aspirin. Patients with concomitant heart failure (HF) are a subgroup with a higher risk for ischemic events. Accordingly, we explored the risks and benefits of DPI therapy in a generalizable population of patients with concomitant atherosclerotic cardiovascular disease and HF.

**Methods:**

The **X**arelto plus **A**cetylsalicylic acid **T**reatment patterns and **O**utcomes in patients with **A**therosclerosis (XATOA) registry is a prospective, multicentre registry of patients with either coronary artery or peripheral artery disease that were given DPI therapy. The primary end point was a composite of cardiovascular death, myocardial infarction, or stroke, and the safety outcome was major bleeding. Multivariable logistic regression was performed to assess the association of HF status and ejection fraction (EF) on the outcomes of interest.

**Results:**

Of 5532 participants, 4022 (72.7%) had documentation of HF status. Of those 873 (21.5%) had a history of HF (EF > 40%, 461; EF ≤ 40%, 181, EF unknown, 231). Over a median follow-up of 465 days (interquartile range, 372-576), the primary outcome occurred in 4.9% of participants with HF compared with 2.4% in those without HF (adjusted hazard ratio, 1.57; 95% confidence interval, 1.02-2.41). The safety outcome was similar in patients with and without HF (0.9% vs 1.11%; a hazard ratio, 0.7; 95% confidence interval, 0.31-1.67).

**Conclusions:**

In a generalizable cohort of patients with atherosclerotic disease and HF, the use of DPI therapy is associated with outcomes similar to those observed in recent randomized controlled clinical trials.

## Résumé

**Contexte:**

Les patients atteints d'une maladie coronarienne athéroscléreuse pourraient bénéficier d'un traitement bimodal d'inhibition plaquettaire (TBIP), comprenant rivaroxaban et aspirine. Les patients souffrant d'une insuffisance cardiaque (IC) concomitante constituent un sous-groupe présentant un risque plus élevé d'événements ischémiques. Par conséquent, nous avons étudié les risques et les avantages du TBIP dans une population généralisable de patients souffrant d'une maladie cardiovasculaire athérosclérotique et d'une IC concomitante.

**Méthodes:**

Le registre XATOA (**X**arelto plus **A**cetylsalicylic acid **T**reatment patterns and **O**utcomes in patients with **A**therosclerosis) est un registre prospectif et multicentrique de patients souffrant d'une maladie coronarienne ou d'une maladie artérielle périphérique qui ont reçu un TBIP. Le critère d'évaluation principal était un composite de décès cardiovasculaire, d'infarctus du myocarde ou d'accident vasculaire cérébral, et le critère d'évaluation de la sécurité était l'apparition d'hémorragies majeures. Une régression logistique multivariable a été réalisée pour évaluer l'association entre l'état de l'IC et la fraction d'éjection (FE) et les critères d'évaluation.

**Résultats:**

Sur 5 532 participants, 4 022 (72,7 %) avaient des informations documentées sur le statut de leur IC. Parmi ceux-ci, 873 (21,5 %) avaient des antécédents d'IC (461 avec une FE > 40 % ; 181 avec une FE ≤ 40 % ; 231 avec une FE inconnue). Au cours d'un suivi médian de 465 jours (intervalle interquartile, 372-576), le critère d'évaluation principal est survenu chez 4,9 % des participants atteints d'IC, contre 2,4 % chez ceux qui n'en étaient pas atteints (rapport de risque ajusté, 1,57 ; intervalle de confiance à 95 %, 1,02-2,41). Le critère d'évaluation de la sécurité était similaire chez les patients avec et sans IC (0,9 % contre 1,11 % ; rapport de risque ajusté, 0,7 ; intervalle de confiance à 95 %, 0,31-1,67).

**Conclusions:**

Dans une cohorte généralisable de patients atteints de maladie coronarienne athéroscléreuse et d'IC, l'utilisation d'un TBIP est associée à des résultats similaires à ceux observés récemment dans des essais cliniques randomisés et contrôlés.

Worldwide, atherosclerotic cardiovascular (CV) disease (ASCVD) is the leading cause of morbidity and mortality, and affects an estimated 126 million individuals worldwide.^1^ Compared with the general population, individuals with established ASCVD are at a higher risk of death, and ischemic cardiac, cerebral, and peripheral vascular events. Cohort studies have shown that 1 of 20 patients with ASCVD will experience a major adverse cardiac event within a year and 1 of 10 within 4 years.^2^^,^^3^ To address this risk of secondary CV events, there have been renewed efforts to enhance secondary prevention therapies through the application of guideline-directed medical therapy.^4^^,^^5^

One secondary prevention strategy is the use of dual pathway inhibition (DPI) therapy with aspirin and vascular-dose rivaroxaban (2.5 mg twice daily), which simultaneously targets the platelet-activation and thrombin-generation pathways. In the Cardiovascular Outcomes for People Using Anticoagulation Strategies (COMPASS) trial, the DPI therapy arm showed an absolute risk reduction of 1.3 without a significant increase in major bleeding compared with the aspirin monotherapy arm.^6^ Although clinical trials provide crucial estimates of the risks and benefits of novel therapeutics, they are often limited by a lack of generalizability, and frequently include a lower-risk population that is younger and has fewer comorbidities.^7^

Several subgroups of ASCVD patients show a greater risk of adverse CV events and have also shown a greater degree of benefit with DPI therapy, which include the presence of concomitant heart failure (HF).^8^, ^9^, ^10^ In the COMPASS trial, participants with HF showed similar relative risk reductions with the use of DPI therapy compared with aspirin monotherapy in those without HF, but showed a larger absolute risk reduction because of higher event rates.^11^ Because of known differences in the characteristics of participants enrolled in clinical trials and those managed in clinical practice, we used the **X**arelto plus **A**cetylsalicylic acid **T**reatment patterns and **O**utcomes in patients with **A**therosclerosis (XATOA) registry^12^ to explore outcomes of DPI in routine clinical practice in patients with and without HF.

## Methods

### Patient population

The XATOA registry is a prospective, international, cohort of 5808 patients older than the age of 18 years with ASCVD (coronary artery disease and/or peripheral artery disease) who were given DPI therapy with rivaroxaban 2.5 mg twice daily in addition to aspirin within 4 weeks of enrollment between November 2018 and May 2020. All patients provided written informed consent. The design of the study and baseline characteristics have previously been reported.^12^^,^^13^ Exclusion criteria included: active clinically significant bleeding, significant risk factors for major bleeding, ongoing treatment with dual antiplatelet therapy for a recent acute coronary syndrome, stroke or transient ischemic attack, participation in an interventional trial, or use of anticoagulation for alternative indications. Patients were recruited from the outpatient and in-hospital setting and followed-up during routine outpatient care. All participants provided informed consent. The enrolling investigator was responsible for determining the presence or absence of all comorbidities including HF and documenting the most recent ejection fraction (EF), if available. The full analysis cohort consisted of all patients who received at least 1 dose of DPI therapy (aspirin and rivaroxaban) and the safety analysis cohort was defined as patients who received at least 1 dose of rivaroxaban (but not aspirin). Indications for inclusion in the safety analysis cohort, but not the full analysis cohort, included violation of inclusion or exclusion criteria, not having received a dose of aspirin and a creatinine clearance decrease to ≤ 30 mL/min within 30 days of rivaroxaban therapy initiation.

### Study outcomes

The primary clinical end point was the composite of major adverse cardiac events (MACE), which includes CV death, myocardial infarction (MI), or stroke. Secondary outcomes included individual components of MACE, major adverse limb events, which includes a composite of acute, severe, or chronic limb ischemia requiring intervention and major amputation above the forefoot. All events were centrally adjudicated by an independent, external adjudication committee. Additionally, HF hospitalization and the composite of HF hospitalization and CV death were assessed. The primary safety end point was major bleeding as defined by the International Society on Thrombosis and Haemostasis (ISTH), which includes fatal or symptomatic bleeding into a critical organ, the need for transfusion of ≥ 2 units of packed red blood cells or whole blood, or a ≥ 2 g/dL reduction in hemoglobin.^14^ Additionally, net clinical benefit was calculated as a composite of MI, stroke, CV death, bleeding in a critical organ, and fatal bleeding. Safety outcomes and net clinical benefit were determined only from patients in the safety analysis cohort, who received at least 1 dose of rivaroxaban.

### Statistical analysis

Continuous variables are summarized as mean ± standard deviation) or otherwise presented as median (interquartile range [IQR]). Categorical variables are summarized as frequencies and percentages. Differences in baseline demographic characteristics between patients with and without HF were analyzed using Wilcoxon 2-sample test for continuous variables and Pearson χ^2^ test without continuity correction for dichotomous or ordinal variables. To assess differences in outcomes between patients with and without HF, confidence intervals for incidence rate of outcomes were calculated on the basis of Poisson distribution. A multivariable Cox regression was undertaken to determine the differences in outcomes between the HF and no HF cohort, as well as according to EF categories (> 40%, ≤ 40%), and per 5% increase, adjusted for sex, age, body mass index, smoking, baseline medication use, and a history of acute coronary syndrome, dyslipidemia, hypertension, type 2 diabetes, stroke, and a family history of premature vascular disease. For models using EF as a continuous variable, EF > 40% was used as the reference category. Hazard ratios, corresponding 95% confidence interval, and Wald type 3 *P* values were computed, Firth penalized maximum likelihood estimates were computed to handle data separation issues (ie, all events occurred only in 1 category of a binary covariate). With the exception of net clinical benefit and major bleeding outcomes, all analyses were conducted in the full analysis cohort.

## Results

### Patient population

The XATOA registry included 5532 patients who received at least 1 dose of rivaroxaban as part of a DPI treatment strategy. Of those, 4022 (72.7%) had their HF status documented and are included in the full analysis cohort. From this population, 873 (21.5%) were documented to have a previous history of HF. Forty-four patients were excluded from the full analysis cohort, but because they had received at least 1 dose of rivaroxaban, were included in the safety analysis cohort. Participants were followed for a median of 465 (IQR, 372-576) days and 79.3% had > 12 months of follow-up. The HF cohort had a median age of 69.2 (IQR, 63.0-75.4) years and 23.9% were female (Table 1). Compared with participants without HF, those with HF had a higher burden of polyvascular disease, previous MI, hypertension, and type 2 diabetes. Most patients (57.3%) reported New York Heart Association (NYHA) class II symptoms, followed by NYHA class III (19.7%), NYHA class I (19.6%), and NYHA class IV (1.1%). EF was documented in 73.5% of cases (n = 642), with 181 (20.7%) patients with an EF of *≤ 40% and 461 (52.8%) with an EF of > 40%.*

**Table 1:** Demographic characteristics stratified according to heart failure status

	Heart failure (n = 873)	No heart failure (n = 3149)
Demographic characteristics		
Age		
Mean years ± SD	69.07 ± 9.03	68.09 ± 9.70
Median years (IQR)	69.26 (63.02-75.35)	68.72 (61.71-75.28)
Sex (% female)	209 (23.9)	688 (21.8)
Race		
White	810 (92.8)	2558 (81.2)
Black or African American	2 (0.2)	9 (0.3)
Asian	30 (3.4)	293 (9.3)
Indigenous	2 (0.2)	32 (1.0)
Not reported	18 (2.1)	222 (7.0)
Mixed ancestry	11 (1.3)	35 (1.1)
Region		
Asia Pacific	10 (1.1)	124 (3.9)
Eastern Europe	326 (37.3)	209 (6.6)
Middle East	65 (7.4)	144 (4.6)
Latin America	27 (3.1)	204 (6.5)
Western Europe and Canada	445 (51.0)	2468 (78.4)
Comorbidities and risk factors		
CAD	435 (49.8)	1839 (58.4)
CAD and PAD	438 (50.2)	1310 (41.6)
Previous MI	513 (58.8)	1492 (47.4)
Dyslipidemia	613 (70.2)	2452 (77.9)
Hypertension	783 (89.7)	2594 (82.4)
Type 2 diabetes	382 (43.8)	1192 (37.9)
Ischemic stroke	58 (6.6)	166 (5.3)
BMI		
Mean ± SD	29.08 ± 5.00	28.47 ± 4.96
Median (IQR)	28.40 (25.69-31.80)	27.77 (25.06-31.10)
FH of premature vascular disease∗	284 (32.5)	997 (31.7)
Smoking		
Never	348 (39.9)	1097 (34.8)
Former	364 (41.7)	1446 (45.9)
Current	161 (18.4)	597 (19.0)
NYHA classification		
NYHA I	171 (19.6)	–
NYHA II	500 (57.3)	–
NYHA III	172 (19.7)	–
NYHA IV	10 (1.1)	–
Mean % ± SD	49 ± 12.4	–
Median % (IQR)	50 (40-60)	–
≤ 40%	181 (20.7)	–
> 40%	461 (52.8)	–
Missing	231 (26.5)	–
Medications		
β-Blockers	705 (80.8)	2156 (68.5)
ACEi/ARB	685 (78.5)	2325 (73.8)
ARNi	39 (4.5)	24 (0.8)
MRA	214 (24.5)	159 (5.0)
SGLT2i	58 (6.6)	247 (7.8)
Statin	765 (87.5)	2704 (85.9)
Treatment for diabetes	289 (33.1)	940 (29.9)
Diuretic	445 (51.1)	785 (24.9)
Protein pump inhibitor	238 (27.3)	907 (28.8)

Data are presented as n (%) except where otherwise noted.

ACEi, angiotensin converting enzyme inhibitor; ARB, angiotensin II receptor blocker; ARNi, angiotensin receptor neprilysin inhibitor; BMI, body mass index; CAD, coronary artery disease; FH, family history; IQR, interquartile range; MI, myocardial infarction; NYHA, New York Heart Association; PAD, peripheral artery disease; SD, standard deviation; SGLT2i, sodium glucose cotransporter-2 inhibitor.

∗ Men younger than 55 years and women younger than 65 years.

### Indications and use of DPI therapy

The most common reasons for initiation of DPI therapy in the HF and no HF cohort were because of perceived high ischemic risk (91.5% vs 83.1%, respectively) and completion of dual antiplatelet therapy (10.8% vs 15.9%, respectively). The mean overall DPI treatment duration in the HF cohort was 437.1 ± 177.9 (median 443; IQR, 371-550) days. In the no HF cohort the DPI duration was similar with a mean of 450.0 ± 202.37 (median 470; IQR, 365-583) days. There was no important difference in the proportion of patients of patients who permanently discontinued DPI therapy (HF, 19.6%; no HF, 21.9%). Of the remaining, the proportion of patients without interruption to their DPI therapy was similar in the HF and no HF cohorts (92.2% vs 92.3%).

### Clinical events

**Figure 1:**
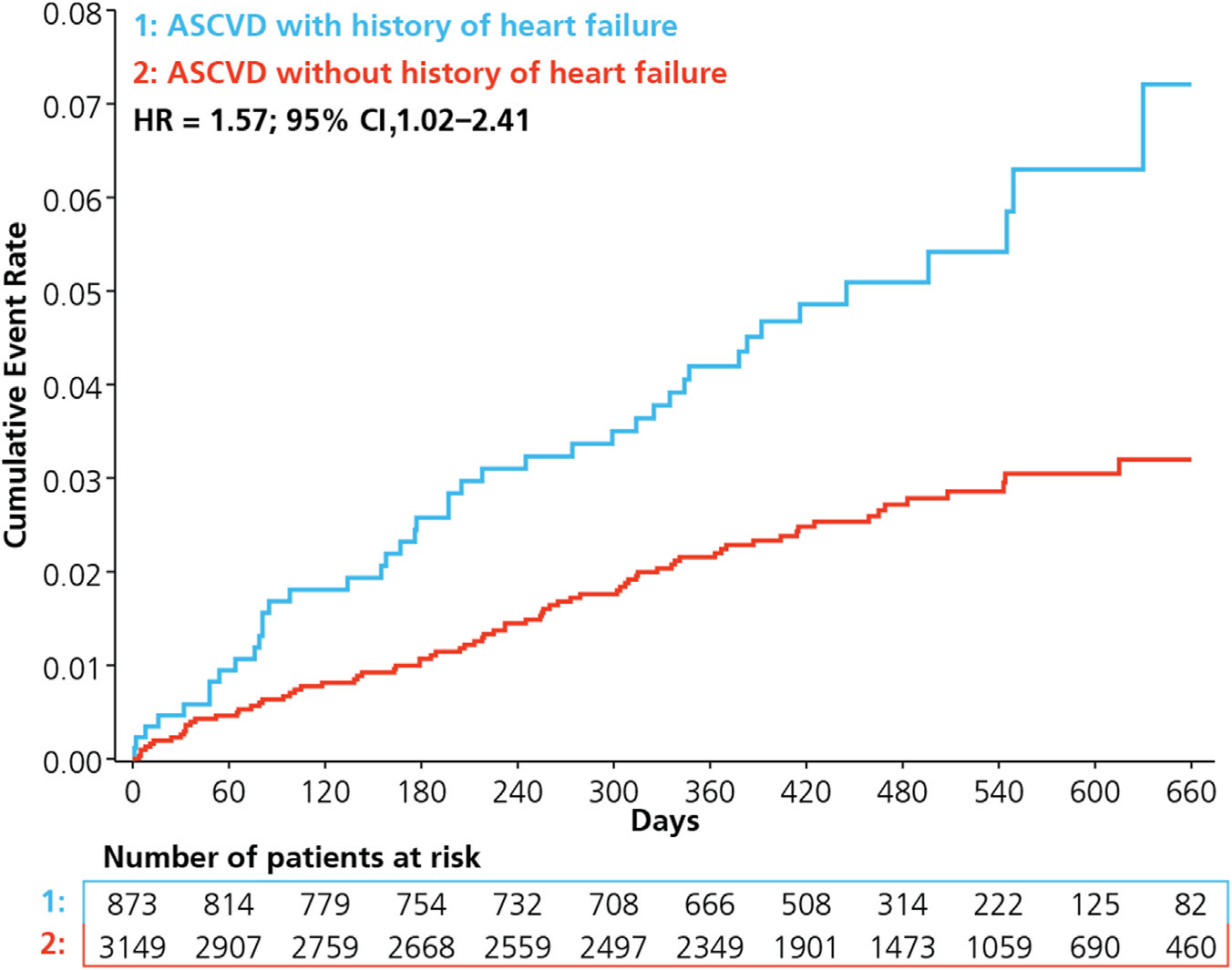
Cumulative incidence of major adverse cardiovascular events stratified according to heart failure status. ASCVD, atherosclerotic cardiovascular disease; CI, confidence interval; HR, hazard ratio.

**Figure 2:**
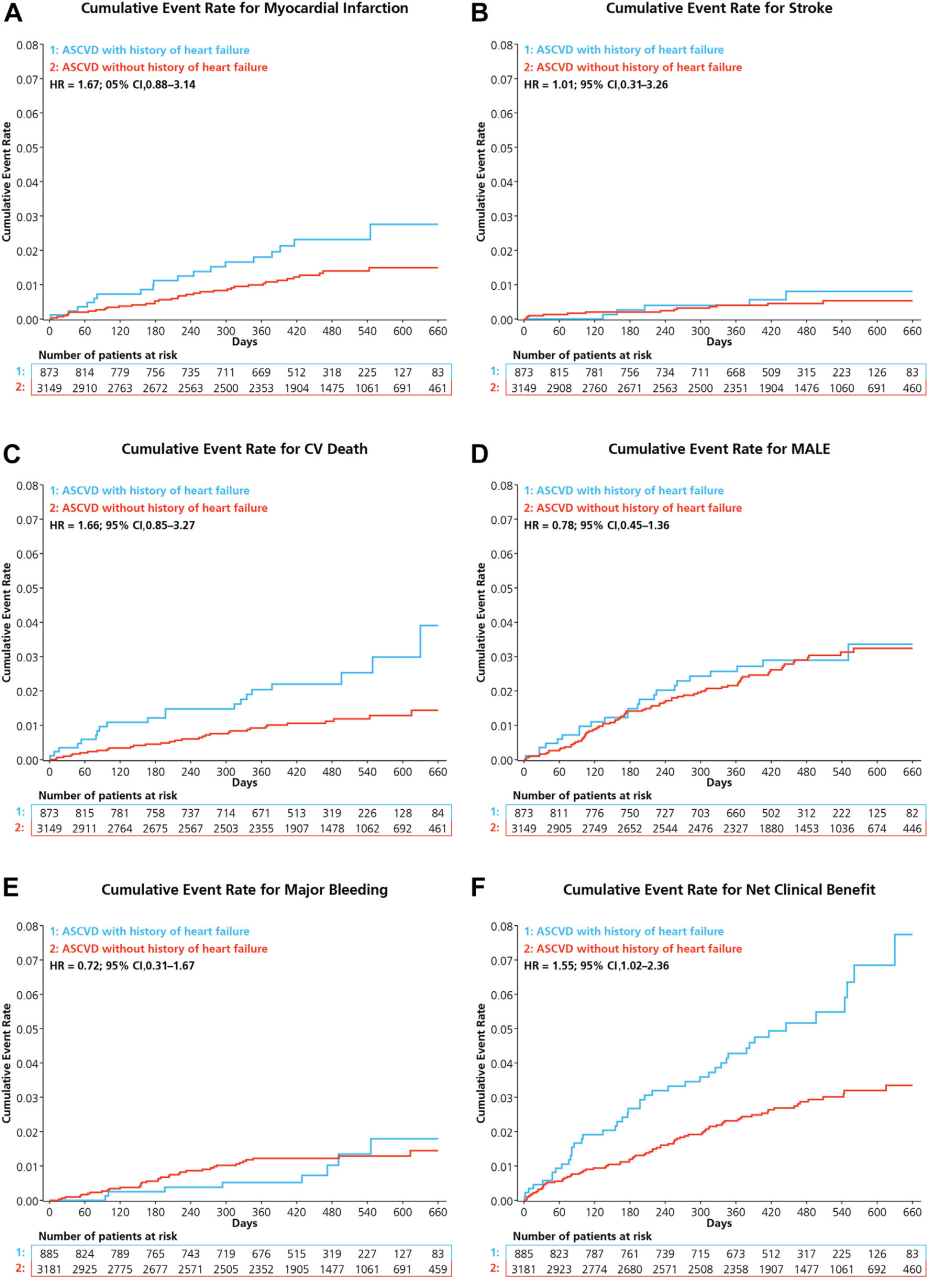
Cumulative incidence of (**A**) myocardial infarction, (**B**) stroke, (**C**) cardiovascular (CV) death, (**D**) major adverse limb events (MALE), (**E**) major bleeding, and (**F**) net clinical benefit. ASCVD, atherosclerotic cardiovascular disease.

During the study period, 4.9% (n = 43) of the HF cohort experienced a major adverse coronary event compared with 2.4% (n = 77) in those without HF, with an adjusted hazard ratio (aHR) of 1.57 (95% confidence interval [CI], 1.02-2.41; Table 2; Fig. 1). This was driven by a higher number of MIs (2.2% vs 1.2%; aHR, 1.67; 95% CI, 0.88-3.14) and CV deaths (2.3% vs 1.0%; aHR, 1.66; 95% CI, 0.85-3.27; Fig. 2). A similar rate of strokes (0.6% vs 0.4%) and major adverse limb events (2.6% vs 2.5%) was observed in the HF and no HF cohorts, respectively. Few hospitalizations for HF occurred, with 6 in the HF cohort and 4 in the cohort without HF. During follow-up, 25 participants developed atrial fibrillation leading to DPI discontinuation, with 8 (0.9%) in the HF cohort and 17 (0.5%) in the no HF cohort.

**Table 2:** Outcomes stratified according to heart failure status

	Heart failure (n = 873)		No heart failure (n = 3149)		Adjusted hazard ratio (95% CI)	*P*
	Incidence, n (%)	Incidence rate (95% CI)∗	Incidence, n (%)	Incidence rate (95% CI)∗		
Primary end point						
MACE	43 (4.9)	4.24 (3.07-5.71)	77 (2.4)	2.02 (1.60-2.53)	1.57 (1.02,2.41)	0.040
Secondary end points						
MI	19 (2.2)	1.86 (1.12-2.91)	37 (1.2)	0.97 (0.68-1.34)	1.67 (0.88,3.14)	0.114
Stroke	5 (0.6)	0.49 (0.16-1.15)	14 (0.4)	0.37 (0.20-0.62)	1.01 (0.31,3.26)	0.986
CV death	20 (2.3)	1.96 (1.20-3.02)	32 (1.0)	0.84 (0.57-1.18)	1.66 (0.85,3.27)	0.140
HF hospitalization	6 (0.7)	0.58 (0.21-1.25)	4 (0.1)	0.10 (0.03-0.26)	5.54 (1.16-26.37)	0.032
Composite CV death and HF hospitalization	26 (3.0)	2.49 (1.63-3.65)	34 (1.1)	0.86 (0.60-1.20)	2.10 (1.13-3.90)	0.019
MALE	23 (2.6)	2.28 (1.44-3.42)	79 (2.5)	2.09 (1.66-2.61)	0.78 (0.45-1.36)	0.381
Major bleeding†	8/885 (0.9)	0.78 (0.34-1.53)	36/3181 (1.1)	0.94 (0.66-1.30)	0.72 (0.31-1.67)	0.448
Net clinical benefit†	45/885 (5.1)	4.39 (3.20-5.88)	82/3181 (2.6)	2.14 (1.71-2.66)	1.55 (1.02-2.36)	0.039

*P* values are from the Wilcoxon 2-sample test for continuous variables, and Pearson χ^2^ test without continuity correction for dichotomous or ordinal variables.

CI, confident interval; CV, cardiovascular; HF, heart failure; MACE, major adverse cardiovascular events; MALE, major adverse limb events; MI, myocardial infarction.

∗ Incidence rate reported per 100 person-years.

† Bleeding and net clinical benefit analysis is derived from the safety analysis cohort.

Major bleeding occurred with a similar incidence in both groups, and occurred in 8 (0.9%) participants in the HF cohort and 36 (1.1%) in the cohort without HF (aHR, 0.72; 95% CI, 0.31-1.67; Fig. 2). The net clinical benefit composite outcome occurred in 45 (5.1%) of the HF cohort and in 82 (2.6%) of the cohort without HF (aHR, 1.55; 95% CI, 1.02-2.36: Fig. 2).

### Outcomes according to EF status

**Figure 3:**
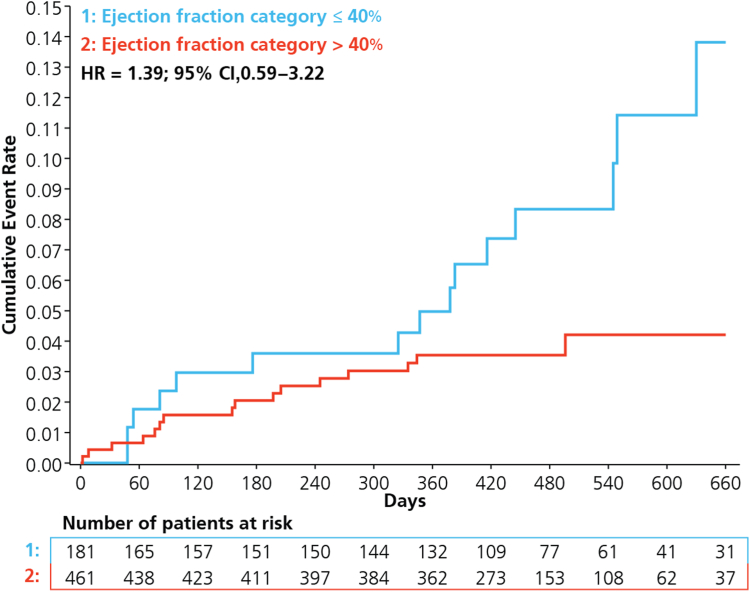
Cumulative event rates for major adverse cardiovascular events according to ejection fraction category ≤ 40% and > 40%. CI, confidence interval; HR, hazard ratio.

Among patients with HF, those with an EF of ≤ 40% showed a trend toward higher rates of adverse events including MACE (8.8% vs 3.5%; aHR 1.38; 95% CI, 0.59-3.22), MI (3.9% vs 1.7%; aHR, 1.30; 95% CI, 0.40-4.23), CV death (4.4% vs 1.5%; aHR, 1.95; 95% CI, 0.49-7.75), and major bleeding (1.6% vs 0.6%; aHR, 2.61; 95% CI, 0.36-19.06; Table 3; Fig. 3). Few stroke events were reported, which limited further analysis. Models that used EF as a continuous variable showed similar results, with a 5% increase in EF associated with decreased risk of MACE (aHR, 0.96; 95% CI, 0.92-0.99; Table 3).

**Table 3:** Outcomes in patients with HF stratified according to EF category

	EF ≤ 40% (n = 181), n (%)	EF > 40% (n = 461), n (%)	Adjusted HR (95% CI), categorized	*P*	Adjusted HR (95% CI), per 5% increase in EF∗	*P*
Primary end point						
MACE	16 (8.8)	16 (3.5)	1.38 (0.59-3.22)	0.4587	0.96 (0.92-0.99)	0.017
Secondary end points						
MI	7 (3.9)	8 (1.7)	1.30 (0.40-4.23)	0.6611	0.97 (0.93-1.02)	0.294
Stroke	2 (1.1)	2 (0.4)	1.23 (0.08-18.09)	0.8803	0.96 (0.87-1.07)	0.475
CV Death	8 (4.4)	6 (1.5)	1.95 (0.49-7.75)	0.3418	0.93 (0.88-0.99)	0.018
HF hospitalization	3 (1.7)	2 (0.4)	5.33 (0.50-56.71)	0.1655	0.94 (0.86-1.02)	0.114
Composite CV death and HF hospitalization	11 (6.1)	8 (1.7)	2.43 (0.74-8.01)	0.1442	0.93 (0.89-0.98)	0.004
MALE	7 (3.9)	11 (2.4)	2.22 (0.60-8.27)	0.2337	0.97 (0.91-1.02)	0.194
Major bleeding†	3/185 (1.6)	3/464 (0.6)	2.61 (0.36-19.06)	0.3445	0.97 (0.90-1.06)	0.541
Net clinical benefit†	17/185 (9.2)	17/464 (3.7)	1.45 (0.64-3.31)	0.3764	0.96 (0.92-0.99)	0.010

CI, confidence interval; CV, cardiovascular; EF, ejection fraction; HF, heart failure; HR, hazard ratio; MACE, major adverse cardiovascular events; MALE, major adverse limb events.

∗ EF > 40% used as the reference category.

† Bleeding and net clinical benefit analysis is derived from the safety analysis cohort.

## Discussion

Patients with ASCVD and concomitant HF who are treated with DPI therapy show twice the rate of MACE, driven by an increase in MI and CV death, compared with those without HF. Treatment with DPI therapy was not associated with an increased risk of major bleeding between patients with and without HF.

Historical trials that examined the use of vitamin K antagonists in patients with HF without evidence of atrial fibrillation, showed a lack of clinical benefit and increased risk of bleeding.^15^ In the COMPASS trial, participants with chronic coronary artery disease or peripheral artery disease were randomized to rivaroxaban 2.5 mg twice daily with 100 mg of aspirin, rivaroxaban 5 mg twice daily, or aspirin 100 mg alone. In the trial, 5902 patients had a history of HF, of whom 12 had an EF of ≤ 40% with EF ≤ 30% as an exclusion criterion. This substudy showed that although patients with and without HF showed a similar relative risk reduction for MACE, there was a considerably larger absolute risk reduction of 2.4% in the HF cohort and 1.0% in the no HF cohort.^11^ They also showed no significant difference in major bleeding among patients with or without HF (3.3% vs 1.9%).

The treatment arm of the COMPASS trial is the only relevant comparator to the presented registry study, which includes a similar population of patients with stable ASCVD (Table 4). Among patients with HF in the XATOA registry, we observed a similar rate of MACE compared with patients with HF in the COMPASS trial (5.0% over 15 months vs 5.5% over 23 months) and a lower risk of major bleeding (0.9% vs 2.5%; Table 4). This is despite that the XATOA registry included more symptomatic HF patients, a greater proportion of patients with an EF ≤ 40%, and those with more comorbidities. The results of the present XATOA registry analysis and subgroup analysis of the COMPASS trial show that patients with an EF ≤ 40% who are receiving DPI therapy have a higher rate of MACE compared with those with an EF > 40% (COMPASS: EF ≤ 40% = 10.2%; EF > 40% = 4.8%; XATOA: EF ≤ 40% = 8.8%, EF > 40% = 3.5%). Both studies showed no significant differences in the rate of major bleeding between EF cohorts.

**Table 4:** Comparison of outcomes in studies of HF patients receiving DPI therapy

	XATOA HF cohort (N = 873)	COMPASS (N = 236)
Study type	Prospective observational cohort	RCT substudy
Population	Patients with CAD or PAD and history of HF	Chronic CAD or PAD with a history of HF (EF ≤ 40%)
Concomitant antiplatelet therapies	Any antiplatelet, 94%	Aspirin, 100%
Follow-up (median)	15.5 Months	23 Months
MACE, n (%)	43 (4.9)	24 (10.2)
CV death, n (%)	20 (2.3)	16 (6.8)
Stroke, n (%)	5 (0.6)	5 (2.1)
MI, n (%)	19 (2.2)	6 (2.5)
HF hospitalization, n (%)	6 (0.7)	16 (6.8)
Major bleeding, n (%)	8 (0.9)	11 (4.7)

CAD, coronary artery disease; COMPASS, Cardiovascular Outcomes for People Using Anticoagulation Strategies; CV, cardiovascular; DPI, dual pathway inhibition; EF, ejection fraction; HF, heart failure; MACE, major adverse cardiovascular events; MI, myocardial infarction; PAD, peripheral artery disease; RCT, randomized controlled trials; XATOA, **X**arelto plus **A**cetylsalicylic acid **T**reatment patterns and **O**utcomes in patients with **A**therosclerosis.

Among all registry-based observational studies, there is the risk of selection bias. However, the XATOA registry included more symptomatic patients with HF and a higher burden of comorbidities compared with those in the COMPASS trial, suggestive against the presence of a significant selection bias. However, it is important to highlight that those in the COMPASS trial and XATOA registry are patients with ASCVD who initiate DPI therapy. This population varies significantly from traditional HF cohorts, which exclude patients with an alternative indication for anticoagulation such as atrial fibrillation and have more stable HF. This makes it challenging to contrast our cohort with other HF cohorts and partially explains why a paucity of HF events were observed. Compared with clinical trials, observational cohorts such as XATOA are at risk of lower ascertainment of bleeding events. Although this might lead to an underestimation of bleeding results in the overall cohort, it is less likely to detract from an analysis comparing subgroups in the cohort. Additionally, pragmatic cohorts without event and/or clinical adjudication are susceptible to higher rates of missing data (such as EF). Despite a nearly equal distribution of ASCVD in men and women, women are under-represented in the XATOA registry (21.8%), highlighting the continued need for focused efforts to improve diversity in CV research.^16^ Our results should be interpreted in the context of these limitations.

Overall, we showed that in a generalizable population of patients with coronary artery disease or peripheral artery disease and concomitant HF, the rates of adverse clinical events and bleeding are similar to those in recent clinical trials. Although patients with an EF of ≤ 40% who are receiving DPI therapy have higher rates of CV events with DPI therapy compared with those with an EF > 40%, previous studies have suggested they might also receive a greater benefit from the use of DPI therapy. On the basis of the cumulative experience from the current study and previous trials, DPI therapy should be strongly considered in patients with ASCVD and concomitant HF.
